# Design and Immunological Validation of *Macaca fascicularis* Papillomavirus Type 3 Based Vaccine Candidates in Outbred Mice: Basis for Future Testing of a Therapeutic Papillomavirus Vaccine in NHPs

**DOI:** 10.3389/fimmu.2021.761214

**Published:** 2021-10-28

**Authors:** Patrick Neckermann, Ditte Rahbaek Boilesen, Torsten Willert, Cordula Pertl, Silke Schrödel, Christian Thirion, Benedikt Asbach, Peter Johannes Holst, Ralf Wagner

**Affiliations:** ^1^ Institute of Medical Microbiology & Hygiene, Molecular Microbiology (Virology), University of Regensburg, Regensburg, Germany; ^2^ Centre for Medical Parasitology, the Panum Institute, Department of Immunology and Microbiology, University of Copenhagen, Copenhagen, Denmark; ^3^ InProTher APS, Copenhagen, Denmark; ^4^ SIRION Biotech GmbH, Munich, Germany; ^5^ Institute of Clinical Microbiology and Hygiene, University Hospital Regensburg, Regensburg, Germany

**Keywords:** MfPV3, HPV, therapeutic vaccine, adenoviral vector, immunogen design, DNA vaccine, invariant chain

## Abstract

Persistent human papillomavirus (HPV) infections are causative for cervical neoplasia and carcinomas. Despite the availability of prophylactic vaccines, morbidity and mortality induced by HPV are still too high. Thus, an efficient therapy, such as a therapeutic vaccine, is urgently required. Herein, we describe the development and validation of *Macaca fascicularis* papillomavirus type 3 (MfPV3) antigens delivered *via* nucleic-acid and adenoviral vectors in outbred mouse models. Ten artificially fused polypeptides comprising early viral regulatory proteins were designed and optionally linked to the T cell adjuvant MHC-II-associated invariant chain. Transfected HEK293 cells and A549 cells transduced with recombinant adenoviruses expressing the same panel of artificial antigens proved proper and comparable expression, respectively. Immunization of outbred CD1 and OF1 mice led to CD8^+^ and CD4^+^ T cell responses against MfPV3 antigens after DNA- and adenoviral vector delivery. Moreover, *in vivo* cytotoxicity of vaccine-induced CD8^+^ T cells was demonstrated in BALB/c mice by quantifying specific killing of transferred peptide-pulsed syngeneic target cells. The use of the invariant chain as T cell adjuvant enhanced the T cell responses regarding cytotoxicity and *in vitro* analysis suggested an accelerated turnover of the antigens as causative. Notably, the fusion-polypeptide elicited the same level of T-cell responses as administration of the antigens individually, suggesting no loss of immunogenicity by fusing multiple proteins in one vaccine construct. These data support further development of the vaccine candidates in a follow up efficacy study in persistently infected *Macaca fascicularis* monkeys to assess their potential to eliminate pre-malignant papillomavirus infections, eventually instructing the design of an analogous therapeutic HPV vaccine.

## Introduction

Despite efficient prophylactic vaccines and the possibility to screen for cervical lesions, infection with human papillomavirus (HPV) was still responsible for more than 340.000 cervical cancer-related deaths worldwide in 2020 (1). More than 85% of these cases occurred in middle- and low-income countries (2). Currently, there are three approved prophylactic vaccines providing near complete protection against vaccine-targeted HPV types, yet vaccine uptake is incomplete (3). However, these vaccines do not lead to the eradication of pre-infected cells, since they target the major capsid protein L1, which is not expressed in infected basal layer- and cervical cancer cells (4–7). Thus, novel interventions such as therapeutic vaccines are desirable.

Whereas most HPV infections are spontaneously cleared within months, some persist for years (8). These can progress towards low-grade squamous intraepithelial lesions (LSIL), which can further progress to high-grade squamous intraepithelial lesions (HSIL) and cervical cancer. The expression pattern of viral proteins changes during progression of SILs: in LSIL, mainly the early proteins E1/E2 are expressed, whereas in HSIL and transformed cells, E6/E7 are highly expressed and E2 expression is low or absent (9, 10).

In many cases of SIL, however, natural regression occurs as a result of CD4^+^ and CD8^+^ T cell infiltration (11). It has been shown that CD4^+^ T cells play a major role in lesion regression with increased CD4:CD8 ratios being found in the stroma of LSIL (12). Genetic studies have shown that resistance and susceptibility loci for chronic infections and cancer cluster in the MHC II region. Cellular immune responses against E1 have been detected in some patients with HPV^+^ cervical squamous cell carcinoma, mostly at low magnitude, but strikingly correlating with improved clinical outcomes (13). Conversely, HPV16^+^ cervical cancer patients have impaired memory CD4^+^ T-helper responses against E2 and E6, which emphasizes the important role of T cell responses in preventing progression and clearing lesions (14–17). It was also reported that HPV-exposed children have E2-specific T cell responses after clearing the infection (18). Current research on therapeutic HPV vaccines is primarily focused on the HSIL and cancer-stage of the disease, and directed toward the oncogenic viral proteins E6 and E7 (7). However, targeting the infection prior to carcinogenesis could be advantageous in terms of reducing morbidity and suffering related to cancer treatment, and might be easier to achieve. Thus, to target pre-malignant infection, other early proteins should be included as antigens.

There is no suitable small animal model to study persistent HPV infections in a preclinical setting, but *Macaca fascicularis* papillomavirus type 3 (MfPV3) has a close phylogenetic and phenotypic relationship to HPV16 (15, 19). Naturally occurring infections with this virus are associated with long-term persistence and at least LSIL-like lesions in the cervix of breeding female cynomolgus macaques (*Macaca fascicularis*), making them an ideal non-human primate (NHP) animal model (20, 21).

In a previous study using recombinant adenovirus-(rAd)-vectored vaccines encoding ancestral E1 and E2 antigens targeting the most conserved N- and C-terminal domains, we observed strong immune responses in mice and in cynomolgus macaques against these proteins, but cross-reactivity against prevalent MfPV types was only observed in a subset of animals. Nevertheless, 3 out of 3 animals with T-cell responses towards MfPV3-E1/E2 ended up clearing the specific MfPV3 infection (22). Furthermore, therapeutic efficacy of both early E1-E2 or E6-E7 antigen DNA vaccines, and synergy from co-administration of both vaccines, was demonstrated in the cottontail rabbit papillomavirus model (23). Combining both approaches by including E1, E2, E6 and E7 in an immunogen to a specific HPV type could potentially target infected cells in all stages of HPV infection and cancer development could be sufficient as therapeutic vaccine. Here, successful stimulation of E1/E2-specific cellular immunity would primarily clear infections in the LSIL-stage (24), whereas E6/E7-specific responses would mainly target the HSIL and cancer stages. As we decided not to attempt a broad ancestral antigen design, we also had the opportunity to include the less conserved parts of full length antigens thereby providing more epitopes for the immune system to act on.

To develop such a therapeutic vaccine, vectors expressing antigen candidates comprising E1, E2, E6 and E7 of MfPV3 were generated and characterized. The antigens are genetically linked to the intrinsic T cell adjuvant MHC-II-associated invariant chain (Ii) that has been shown to increase viral-vector-induced T cell responses in mice, cynomolgus macaques and humans (25–27). The antigens were designed in different configurations as artificial fusion proteins and initially characterized *via* DNA vaccination of outbred CD1 mice. Based on this initial characterization, adenoviral vectors from serotype 19a/64 were generated and characterized *in vitro* as well as *in vivo*.

## Methods

### Antigen Sequences

**Figure 1:**
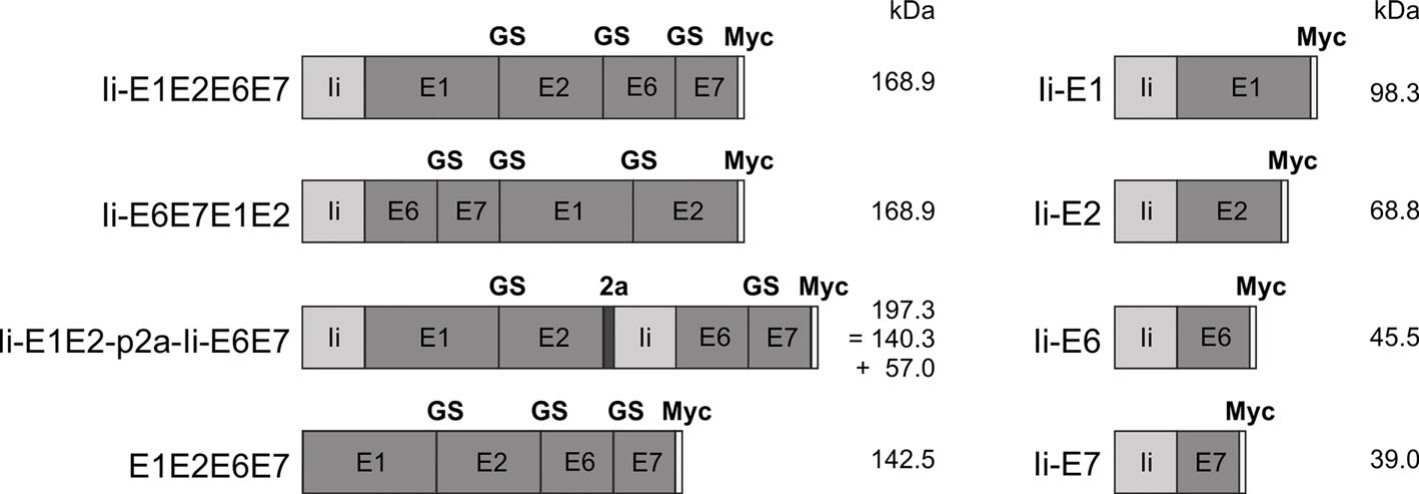
Schematic representation of the conceived MfPV3 antigen variants. MfPV3 antigens were designed as fusion proteins comprising either E1, E2, E6 and E7 altogether, or E1 plus E2 and E6 plus E7 fusion proteins linked by a p2a peptide, and fused to the MHC-II invariant chain (Ii), respectively. For reference, E1, E2, E6, and E7 were fused as single open reading frames without the Ii coding sequence. Calculated molecular weights are indicated (kDa). Ii, MHC-II associated invariant chain; GS, glycine-serine-linker; 2a, p2a peptide for cotranslational separation; Myc, myc-tag sequence for antibody detection; kDa, kilo Dalton.

Parts of the sequences encoding E1, E2, E6, and E7 of MfPV3 (EF558839.2) were designed and synthesized at Geneart/Thermo Fisher (Regensburg, Germany) (28). Mutations were introduced into E6 and E7 to inactivate the oncogenic potential: L110Q and deletion of C-terminal ETEV in E6; E7 was modified by D24G, L71R, C95A; C297A was introduced into E2 to inactivate DNA-binding. Further sequence elements that were optionally used to build the prototype vaccine inserts (**Figure 1**) comprised (i) the human MHC-II-associated Ii invariant chain (25–27) (molecular adjuvant; NM_004355.3), (ii) a 2 amino acid GS-linker connecting E1 and E2 as well as E6 and E7 in the respective fusion proteins as well as (iii) the porcine teschovirus-1 p2A sequence (29) to support co-expression of two fusion proteins, respectively. Vaccine inserts were assembled either with fusion PCR, or using type IIs exocutter sites (“Golden gate” cloning; BsaI-HF v2, New England Biolabs, Ipswich, USA) or the NEBuilder HIFI DNA Assembly Kit (New England Biolabs, Ipswich, USA) according to manufacturer’s instructions, and cloned into pURVac, a derivative of a DNA vaccine vector with a proven track record in various NHP and clinical trials (30–33). Vaccine inserts were then subcloned into pO6-19a-HCMV-MCS for subsequent generation of recombinant adenoviruses (34).

### Cell Lines, Transfection, and Viral Infection

HEK293T cells and A549 cells were maintained and grown in Dulbecco’s MEM (DMEM) supplemented with 10% Fetal Calf Serum (FCS) and 1% Penicillin/Streptomycin (Pen/Strep). 9E10 mycl hybridoma cells were cultivated in RPMI supplemented with 10% FCS, 1% Pen/Strep and 2 mM glutamine (Pan). All cell lines were maintained at 37°C and 5% CO_2_ in a non-humidified incubator. HEK293T cells were transfected using the polyethylenimide (PEI) method (35). For PEI transfection, 4 × 10^5^ cells were seeded in 6-well plates one day before transfection. The cells were transfected with 2.5 µg plasmid (equimolar amounts, filled with empty vector) and 7.5 µg PEI in DMEM without any supplements. After 6 h incubation, medium was exchanged to DMEM with 10% FCS and 1% Pen/Strep.

Subconfluent A549 cells were infected with Ad19a/64 vectors at an MOI of 30 in DMEM without any supplements. 2 h post infection, medium was exchanged to DMEM with 10% FCS and 1% Pen/Strep.

### Generation and Titration of Adenoviral Vectors

E1/E3 deficient adenoviral vectors of serotype Ad19a/64 (rAd) were generated as previously described (34). Briefly, the vaccine inserts (**Figure 1**) were cloned into the shuttle vector pO6-19a-HCMV-MCS under control of a CMV promoter. The resulting plasmids were then inserted *via* Flp-recombination in *E. coli* into a BAC vector containing the genome of a replication deficient Ad-based vector deleted in E1/E3 genes. Recombinant viral DNA was released from the purified BAC-DNA by restriction digest with PacI. The obtained linear DNA was transfected into HEK293T cells for virus reconstitution and propagation. Recombinant viruses were released from cells *via* sodium deoxycholate treatment. Residual free DNA was digested by DNase I. Afterwards, vectors were purified by CsCl gradient ultracentrifugation followed by a buffer exchange to 10 mM Hepes pH 8.0, 2 mM MgCl_2_ and 4% Sucrose *via* PD10 columns (GE Healthcare, Chicago, USA). Titration was performed using the RapidTiter method by detection of infected HEK293T cells *via* immunohistochemical staining with anti-hexon antibody (Novus, Adenovirus Antibody (8C4)). Insert integrity was confirmed by PCR amplification from the purified vector DNA followed by DNA sequencing.

### Antibodies and Antibody Purification

The antibody against myc (9E10) was obtained from hybridoma cell supernatants. 9E10 mycl hybridoma cells were seeded at 5 × 10^5^ cell per ml in RPMI supplemented with 1% FCS, 1% Pen/Strep and 2 mM glutamine. The supernatant was harvested 5 days after seeding and the antibody was purified *via* a HiTrap Protein G column (GE Healthcare, Chicago, USA). After washing the column with PBS, the antibody was eluted with 0.1 M glycine/HCl (pH 3.2), neutralized with 0.025 volumes of 1 M Tris/HCl (pH 9) and dialyzed against PBS.

Other antibodies used were: mouse anti-p2a peptide (3H4, 1:2000, Merck, Darmstadt, Germany), mouse anti-tubulin (DM1α, 1:1000, Santa Cruz, Heidelberg, Germany), mouse anti-ubiquitin-Biotin (eBioP4D1, 1:1000, Invitrogen, Carlsbad, USA), goat anti-mouse-HRP (115-036-003, 1:5000, Jackson, West Grove, USA), goat anti-rabbit-HRP (P0448, 1:2000, Dako, Santa Clara, USA), Streptavidin-HRP (11089153001, 1:5000, Roche, Basel, Swiss), rat anti-mouse-PE (A85-1, 1:100, BD, Franklin Lakes, USA).

### Western Blot Analysis

Western blot analysis was performed as previously described (36). Briefly, cells of interest were lysed in TDLB buffer (50 mM Tris, pH 8.0, 150 mM NaCl, 0.1% SDS, 1% Nonident P-40, 0.5% sodium deoxycholate) supplemented with protease inhibitors (Complete Mini, Roche, Basel, Swiss). Total protein concentration of the supernatants was measured by the Bradford method (Protein Assay, BioRad, Feldkirchen, Germany). The proteins were separated on SDS-PAGE under reducing conditions and blotted on a nitrocellulose membrane for western blot analysis. Targets were probed with primary and secondary antibodies as listed above. HRP-labeled secondary antibodies and enhanced chemiluminescence substrate or Femto ECL (Thermo Fisher, Waltham, USA) were used for detection in a Chemilux Pro device (Intas, Göttingen, Germany).

### Analysis of Ubiquitination

To analyze ubiquitinylated proteins, 24 h post transfection, cells were treated with 10 µM MG132 proteasome inhibitor for 6 h. Afterwards, cells were harvested in PBS and washed twice. For inactivation of deubiquitination enzymes, 20 mM N-ethylmaleimide from a freshly prepared stock solution were added to the TDLB lysis buffer. Lysates were generated as described above. Before immunoprecipitation, Protein G dynabeads (Thermo Fisher, Waltham, USA) were loaded with 10 µg of pulldown antibody. Using these beads, target protein was immunoprecipitated out of 500 µg cell lysate over night at 4°C under slow rotation. After washing the beads four times with PBS, SDS-PAGE buffer was added to the beads before heating at 95°C for 10 min. The samples were used for western blot analyses as described above.

### Flow Cytometry Analysis of Cell Lines

Intracellular staining of antigens was performed using standard methods (36). Cells were fixed and permeabilized with Cytofix/Cytoperm-Buffer (4% PFA, 1% saponine, in PBS). All washing steps were done with Perm/Wash-Buffer (PBS containing 0.1% saponine). The cells were stained with anti-myc antibody (5 µg/ml, diluted in Perm/Wash-Buffer) and rat anti-mouse-PE (1:100 diluted in Perm/Wash-Buffer) each for 30 min. Flow cytometry was performed using an Attune NxT device (Thermo Fisher, Waltham, USA) with a 488 nm excitation and a 574/26 nm emission filter. Cells were gated on stained, mock-transfected cells. Evaluation of data was performed using Attune NxT software.

### Animals and Immunizations

BALB/c and CD1 mice were obtained from Envigo (Horst, The Netherlands) and OF1 mice from Charles River (France). All animals were female, 6-8 weeks old and housed at the Panum Institute, University of Copenhagen. All experiments were initiated after allowing the mice to acclimatize for at least 1 wk. Experiments were approved by the National Animal Experiments Inspectorate (Dyreforsøgstilsynet, license no. 2016-15-0201-01131) and performed according to national guidelines. DNA immunizations were performed intradermal (i.d.) with 0.5 g DNA coated onto 1.6 µm gold microcarriers (BioRad, Feldkirchen, Germany) using the Helios Genegun System (BioRad, Feldkirchen, Germany). The mice received four DNA immunizations at intervals of one week each. One group received a mixture of four plasmids, 0.5 µg each, into one site. Immunizations with adenoviral vectors were performed intramuscular (i.m.) with 2×10^7^ IFU rAd diluted in 50 µL PBS. Mice were anesthetized with isofluorane before rAd injections. One group received a mixture of four rAd, 2×10^7^ IFU each, into one site.

### Flow Cytometry of Splenocytes

Single cell suspensions of splenocytes were obtained by organ harvest in HANKs followed by straining through 70 µm cell strainers. Cells were incubated for 5 hours in 3 µM monensin with or without 1 µg/mL of relevant peptides. The cells were stained against surface markers: APC-Cy7 or BV421 CD8 (53-6.7, 1:200, BioLegend, San Diego, USA), PE-Cy7 CD4 (RM4-5, 1:800, BD), FITC CD44 (IM7, 1:100, BioLegend, San Diego, USA) and PerCP-Cy5.5 B220 (RA3-6B2, 1:200, BioLegend, San Diego, USA). After surface staining, cells were fixed in 1% PFA, permeabilized in 0.5% saponine, and stained intracellularly using APC IFN-γ (XMG1.2, 1:100, BioLegend, San Diego, USA) and PE TNF-α (MP6-XT22, 1:100, BioLegend, San Diego, USA) antibodies. The peptides used were 16-mers overlapping by 11 amino acids covering the entire Ii-E1E2E6E7 antigen. The peptides were pooled in 5 separate pools containing Ii (45 peptides), E1 (123 peptides), E2 (70 peptides, E6 (28 peptides) and E7 (21 peptides) peptides respectively. Peptides were obtained from KareBay, Town, China.

Flow cytometry was performed on the Fortessa 3 (BD Biosciences, Franklin Lakes, USA) flow cytometer and data analysis was performed using FlowJo V10 software. Epitope-specific CD8^+^ T-cell responses were measured as B220^-^, CD8^+^ or CD4^+^, CD44^+^, IFN-γ^+^ cells and are presented in total number of cells per organ. The quality of the IFN-γ^+^ responses were evaluated by MFI of IFN-γ and fraction of double positive cells (expressing both IFN-γ and TNF-α) in the IFN-γ^+^ CD8^+^ or CD4^+^ populations. The gating strategy is shown in **Supplementary Figure 1**.

### *In Vivo* Cytotoxicity

The assay was performed similarly to what was previously described (37). Briefly, splenocytes from naïve BALB/c mice were incubated with MfPV3 E1, E2, E6 or E7 peptide pools (same as used for immune response analyses, see above) or no peptide for 30 minutes at 37°C, 5% CO_2_, 2.5 µg of each peptide/mL, and subsequently stained with combinations of 0.4 or 5 µM CellTrace CFSE and 0.2 and 2.5 µM CellTraceViolet (CTV; ThermoFisher, Waltham, USA) for 10 minutes at 37°C, 5% CO_2_. Pulsed and stained splenocytes were mixed at a 1:1:1:1:1 ratio, and a total of 2.5 x10^7^ cells were injected intravenously into rAd-vaccinated recipient BALB/c mice. As the assay requires adoptive transfer of syngeneic target cells, it was necessary to use inbred mice in order to have HLA-matching. 5 hours later, spleens were harvested, and target cells were identified on the Fortessa 3 (BD Biosciences, Franklin Lakes, USA) flow cytometer by CFSE/CTV staining. The percentage of killing was calculated using the following equation:


% targeted killing=100−(% peptide pulsed cells in vaccinated mice% non−peptide pulsed cells in vaccinated mice% peptide pulsed cells in non−vaccinated mice% non−peptide pulsed cells in non−vaccinated mice)∗100

### Analysis of E1 (SIINFEKL) Presentation on MHC-I

HEK293T cells were transfected with 2.5-3.5 µg of pURVac encoding E1, which was C-terminally extended by the SIINFEKL Ovalbumin derived CD8^+^ T cell epitope and a myc-tag and optionally fused to the Ii T cell adjuvant (25–27) at its N-terminus together with a pUC57 encoding H2Kb and β-2-microglobulin (β2m). A transfection with H2Kb alone was included as a negative control, and transfection with the H2Kb plasmid and a pUC57 plasmid encoding SIINFEKL fused directly to β2m was included as a technical positive control. 48 hours post transfection, cells were stained with PE anti-H2Kb-SIINFEKL (25-D1.16, 1:160, Invitrogen, Carlsbad, USA) and presence of SIINFEKL-H2Kb presentation on cell surfaces was detected on the LSRII or Fortessa 3 (BD Biosciences, Franklin Lakes, USA) flow cytometers, as a proxy for E1 presentation. All samples were run in biological 6-plicates, and the experiment was repeated at least two times.

### Graphical Representation and Statistical Analysis

Non-stimulated samples were used as background controls, and their response values have been subtracted from the peptide-stimulated samples of the corresponding animal before performing statistical analysis and graphical presentation.

In order to aid visual presentation of the results, we applied a threshold for responses based on the average number of B220^-^, CD8^+^, CD44^+^, IFN-γ^+^ counts for unstimulated background samples. All samples with less counts than the average + 2 × SD of the background samples, are regarded as non-responding and therefore they have manually been adjusted to a value of 100 for the graphical presentation. All statistical analyses were done on non-adjusted values. The analysis of fraction of double positive (% TNF-α^+^ IFN-γ^+^ out of all IFN-γ^+^) and MFI of IFN-γ^+^ was only performed on responders (IFN-γ^+^ count > avg + 2 × SD of unstimulated samples).

Statistical analysis was done using GraphPad Prism 8 software (GraphPad Software, San Diego, USA). Values with background subtracted were used to compare the individual groups using the Mann-Whitney test with Bonferroni correction for multiple comparisons. Positive control groups were not included in the multiple comparison analyses. Each symbol represents one mouse. Bars indicate median. Significance levels are marked by * (p<0.05), ** (p<0.01), *** (p<0.005), **** (p<0.001).

## Results

### Design of the Antigens

Aiming towards the generation of a therapeutic vaccine, which is able to eliminate pre-existing papillomavirus-derived premalignant neoplasias, various antigens comprising E1, E2, E6, and E7 were designed. In order to enable future validation of a therapeutic concept in non-human primates, the above antigens were derived from *Macaca fascicularis* papillomavirus type 3 (MfPV3), which was shown to persist and induce LSIL-like lesions in the cervix of breeding female cynomolgus macaques (*Macaca fascicularis*) (20, 21). Herein, we describe the construction and immunological down-selection of eight different antigen designs in an outbred mouse model according to potency and breadth of induced T cell responses as basis for further preclinical validation in non-human primates.

Antigens were conceived as (i) read-through polypeptides with all four MfPV3 early antigens encoded in one open reading frame, (ii) alternatively E1-E2 and E6-E7 fusions were linked *via* a p2A site (38) supporting translational coupling and thus co-expression of the two fusion proteins or (iii) as single expression units. Within fusion proteins, the different MfPV3 early proteins were separated by a GS-linker. With one exception (E1E2E6E7), all polypeptides were NH_2_ terminally fused to a the human MHC class II invariant chain (Ii), which has been described earlier to act as a T cell adjuvant (25). Furthermore, all constructs received a C-terminal myc-tag for convenient expression monitoring (**Figure 1**).

Since E6 and E7 are highly potent oncoproteins, only transformation-defective variants should be used in a therapeutic vaccine setting to ensure safety. Based on the homology to HPV16, the oncoprotein E6 was inactivated by introducing a L110Q substitution to prevent binding of E6 to E6BP and subsequent degradation of tumor suppressor p53 (39). Additionally, the C-terminal PDZ-domain was deleted (ΔETEV) to abolish binding of telomerase and other LxxLL proteins (40). E7 was inactivated by introducing the substitutions C24G, L71R, and C95A within the central LxCxE motif inhibit dimerization and reduce binding to pRB as well as Mi2β (40, 41). Furthermore, we changed E2 (C297A) to reduce DNA binding.

For initial biochemical and immunological characterization, the antigen coding sequences (**Figure 1**) were inserted into pURVac, a derivative of a DNA vaccine vector with a proven track record in various NHP and clinical trials (30–33). Transcription of the encoded transgenes is controlled by a human cytomegalovirus (CMV) promoter in combination with a human T-cell leukemia virus-1 (HTLV-1) regulatory element (42) and terminated by a bovine growth hormone poly-A site.

### Biochemical and Cell Biological Characterization of MfPV3 Antigens

**Figure 2:**
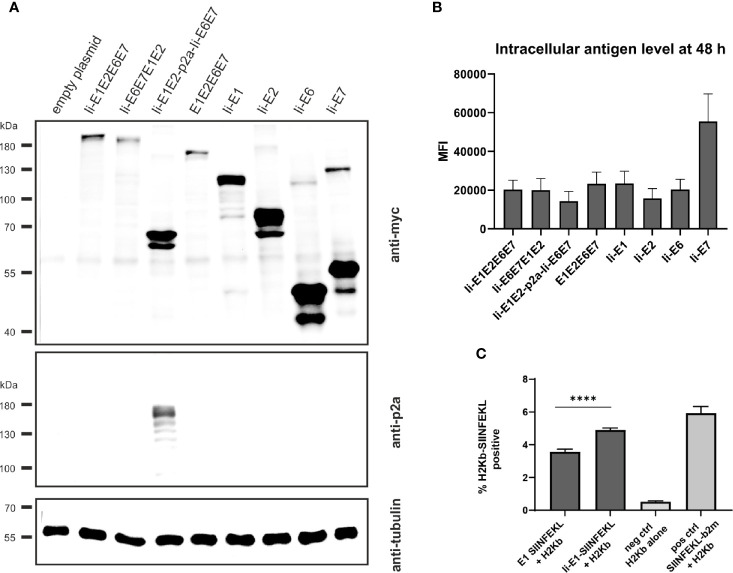
Expression analysis of MfPV3 antigens. **(A)** Western blot analysis of HEK293T cell lysates 48 h following transfection with equimolar amounts of pURVac DNA vaccines expressing the various MfPV3 antigens. Antigens were detected with anti-myc (upper panel) and anti-p2a-peptide (middle panel) antibodies. Tubulin levels were monitored using an anti-tubulin antibody as loading control (lower panel). **(B)** Flow cytometry analysis of HEK293T cells 48 hours following transfection with pURVac DNA vaccines. Intracellular staining was performed with anti-myc antibody. Depicted is the mean fluorescence intensity (MFI) of the average of 3 independent experiments. Error bars indicate standard error of the mean. **(C)** Co-transfection of HEK293T cells with H2Kb alone or together with pURVac E1-SIINFEKL or Ii-E1-SIINFEKL, respectively. The percentage of SIINFEKL-H2Kb positive cells were determined after 48 hours by flow cytometry using an antibody recognizing the SIINFEKL-H2Kb-complex. Error bars indicate standard error of the mean. Light gray bars are negative (no peptide, H2Kb-plasmid only) and positive controls (SIINFEKL-β2m encoding plasmid + H2Kb), respectively. ****p < 0.001.

Following transient transfection of HEK293T cells with the respective DNA vaccine constructs, all antigens were properly expressed yielding proteins chiefly correlating with the calculated molecular weights (**Figure 2A**). In some cases, especially for proteins with high signal intensities, bands of higher or lower molecular weight than calculated were observed. These might result from incompletely reduced oligomers (43) or N-terminal degradation products, which is expected as Ii transports some of its linked cargo to endosomal compartments where proteolytic degradation naturally occurs.

Since proteins of different sizes behave differently during blotting and signal intensities may not reflect the real expression levels, MfPV3 antigens were quantified by flow cytometry after intracellular staining with the anti-myc-antibody (**Figure 2B** and **Supplementary Figure 1**). Protein levels of the different MfPV3 antigens differed only marginally, except for Ii-E7, which exhibited two- to threefold higher MFI values compared to all other antigens.

### MHC Class I-Restricted SIINFEKL Epitope Is Processed From E1 Fusion Protein and Abundantly Presented on MHC-I Molecules *In Vitro*

It has been reported that E1 of bovine papillomavirus type 1 is itself an unstable protein which is ubiquitinylated and rapidly degraded in naturally infected cells (44, 45). This observation together with the reported low prevalence of E1 specific antibodies in women with cervical disease (46) prompted us to assess whether MHC class I-restricted peptides at all can be processed and presented from E1. Therefore, the minimal H2Kb-restricted OVA epitope SIINFEKL (47) was fused to the C-terminus of E1 or, alternatively, to Ii-E1. The SIINFEKL epitope is a high affinity, highly immunodominant epitope, and therefore it is processing and presentation is easy to detect in *in vitro* studies. Following transfection of the respective pURVac expression constructs, MHC class I-restricted presentation of SIINFEKL could be readily detected using an H2Kb-SIINFEKL specific antibody. This supports the choice of E1 as a relevant antigen to be included in the therapeutic vaccine. Noteworthy, presentation was enhanced, when this fusion protein was N-terminally linked to the MHC class II invariant chain (Ii) (**Figure 2C**).

### DNA Vaccination of Antigen Constructs Induces CD4^+^ and CD8^+^ T Cell Responses Against MfPV3 Early Antigens

**Figure 3:**
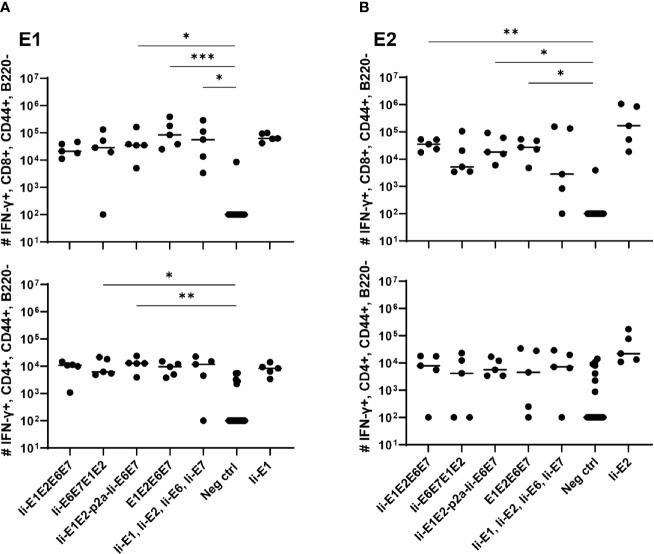
T cell responses induced by the various pURVac DNA vaccines encoding E1, E2, E6 and E7 in outbred CD1 mice. CD1 mice (5 per group) were immunized 4 times in 1 week intervals with 0.5 µg DNA of pURVac DNA encoding the indicated MfPV3 early antigens. Mice were sacrificed 7 days post last immunization, spleens were harvested and CD8^+^ (top panels) and CD4^+^ (bottom panels) T-cell immune responses against E1 **(A)**, and E2 **(B)** were measured using ICS and flow cytometry. Negative control groups consist of all mice immunized with a pURVac DNA vaccine encoding antigens not covered by the peptide pools used for *in vitro* restimulation. Asterisks between groups indicate significant differences in response-levels after subtraction of background responses. Each symbol represents one mouse; the horizontal bar represents the median. Reference samples (mice vaccinated with pURVac DNA vaccine containing only the individual antigen linked to Ii, respectively) are not included in the statistical analysis (multiple comparison adjustment). *p < 0.05; **p < 0.01, ***p < 0.005.

Intradermal (i.d.) DNA immunization of outbred CD1 mice confirmed that all pURVac DNA vaccines encoding E1 and/or E2 induced E1- and E2-specific IFN-γ^+^ CD8^+^ and CD4^+^ T cells (**Figure 3** and **Supplementary Figure 2**). More detailed, all DNA vaccine candidates had provided an E1 response on par with the positive control (Ii-E1). None of these vaccines induced responses against E6 or E7 in these outbred mice, including the positive controls for these two antigens (Ii-E6; Ii-E7; **Supplementary Figures 3A, B**). Importantly, all vaccines encoding all four antigens as a polyprotein generated responses comparable to those induced by a mix of four vaccines encoding the antigens individually (Ii-E1, Ii-E2, Ii-E6, Ii-E7). This suggests that overall no immunogenicity was lost by delivering the MfPV3 early antigens as polyproteins from a single pURVac DNA vaccine. Regardless of the used pURVac vaccine construct, E1 and E2 specific T cell responses showed no statistical differences regarding the frequencies of E1- and E2-specific IFN-γ^+^ CD8^+^ and CD4^+^ T cells. This is the case regardless (i) of whether Ii is fused to E1E2E6E7, (ii) of the order of the early antigens in the polyprotein, and (iii) of the use of a p2a site to separate Ii-E1E2 from Ii-E6E7. Only pURVac-Ii-E2, when administered alone, trended to induce slightly higher levels of specific CD4^+^ and CD8^+^ positive T cells (although statistically not significant). Contrasting previous findings, N-terminal fusion of the Ii molecular adjuvant did not enhance antigen specific T cell responses when delivered as DNA (48, 49). However, there are much fewer examples of Ii having an adjuvant effect in a DNA context compared to when it is encoded in viral vector transgenes. Additionally, it might be that the lack of Ii-effect is due to the high number of DNA immunizations (four in total), as the continuous boosting may have saturated the immune activation. It is possible that a difference between the Ii and non-Ii vaccines could have been detected after fewer immunizations. We were satisfied to see that all DNA constructs were immunogenic, and to address the Ii effect properly, we decided to create adenoviral vectors of all vaccine designs (see below). The quality of the T-cell responses was comparable across all vaccines (**Supplementary Figures 3C, D**), assessed by MFI of IFN-γ and TNF-α production of IFN-γ^+^ T-cells. As expected, we found Ii specific CD8^+^ T cell responses in most mice, and to CD4^+^ in some mice (**Supplementary Figures 3A, B**), as the human Ii is allogenic in mice.

### Characterization of Adenoviral Vectors

**Figure 4:**
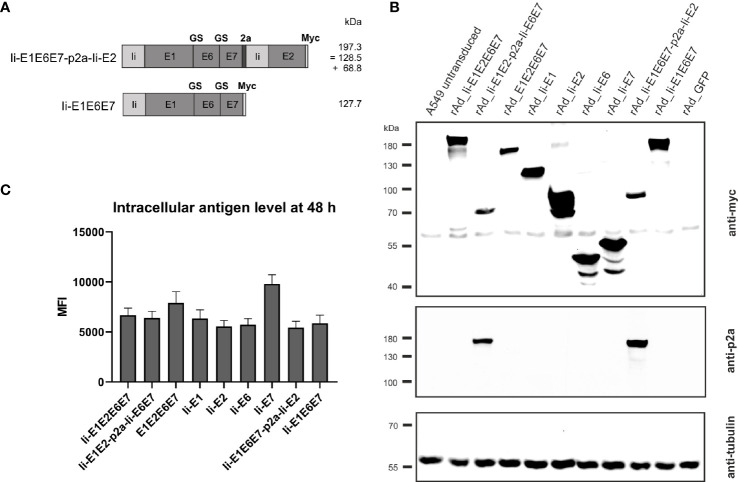
Expression analysis of rAd-shuttled MfPV3 antigen constructs. **(A)** Schematic representation of the MfPV3 antigen variants Ii-E1E6E7-p2a-Ii-E2 and Ii-E1E6E7. Expected molecular weights (kDa) of the resulting polypeptides are shown. **(B)** Western blot analysis of A549 cell lysates 48 h following transduction with rAds encoding the indicated polypeptides at an MOI of 30. Antigens were detected with anti-myc (upper panel) and anti-p2a-peptide (middle panel) antibodies. As loading control, tubulin levels were monitored using an anti-tubulin antibody (lower panel). **(C)** Flow cytometry analysis of A549 cells 48 hours following transduction with rAds expressing the various MfPV3 antigens, at an MOI of 30. Intracellular staining was performed with anti-myc antibody. Depicted is the mean fluorescence intensity (MFI) of the average of 3 independent experiments. Error bars indicate standard error of the mean.

To further increase antigen specific cellular immune responses, viral vectors were subsequently used for antigen delivery. Adenoviral vectors from serotype 19a/64 (rAd) had been shown earlier to be generally suitable to deliver MfPV3 antigens and to efficiently induce CD8^+^ T cell response in cynomolgus macaques (50). Based on the above DNA vaccination data, a refined panel of rAd vectors was generated comprising a modified set of recombinant MfPV3 antigens. Ii-E6E7E1E2 was excluded because it was not superior to the other polyproteins. However, the trend toward higher E2 specific T cell when administered alone prompted us to generate two additional adenoviruses, one encoding Ii-E1E6E7 linked to E2 *via* a self-separating p2a peptide (Ii-E1E6E7-p2a-Ii-E2) and for control Ii-E1E6E7 lacking E2. In order to confirm proper expression of the encoded antigens, western blot analysis was performed with A549 cells following transduction with the indicated rAds. All vectors readily expressed the antigens with bands resembling the respective fusion proteins at the expected molecular weights (**Figures 1** and **4A**, **B**). As for Ii-E1E2-p2a-Ii-E6E7, no higher than calculated molecular weight fragment could be detected for Ii-E1E6E7-p2a-Ii-E2, revealing that the complete separation at the p2a site also worked upon delivery *via* rAds. Quantitative analysis of transduced A549 cells by flow cytometry yielded comparable expression levels for the different myc-tagged MfPV3 polypeptides with the exception of higher expression of Ii-E7 (**Figure 4C** and **Supplementary Figure 4**). The overall picture was quite similar to the expression efficiencies observed after pURVac mediated antigen delivery in HEK293T cells.

### Adenoviral Delivery Induces Potent Cellular Immune Responses Against MfPV3 Early Antigens in Outbred Mice

**Figure 5:**
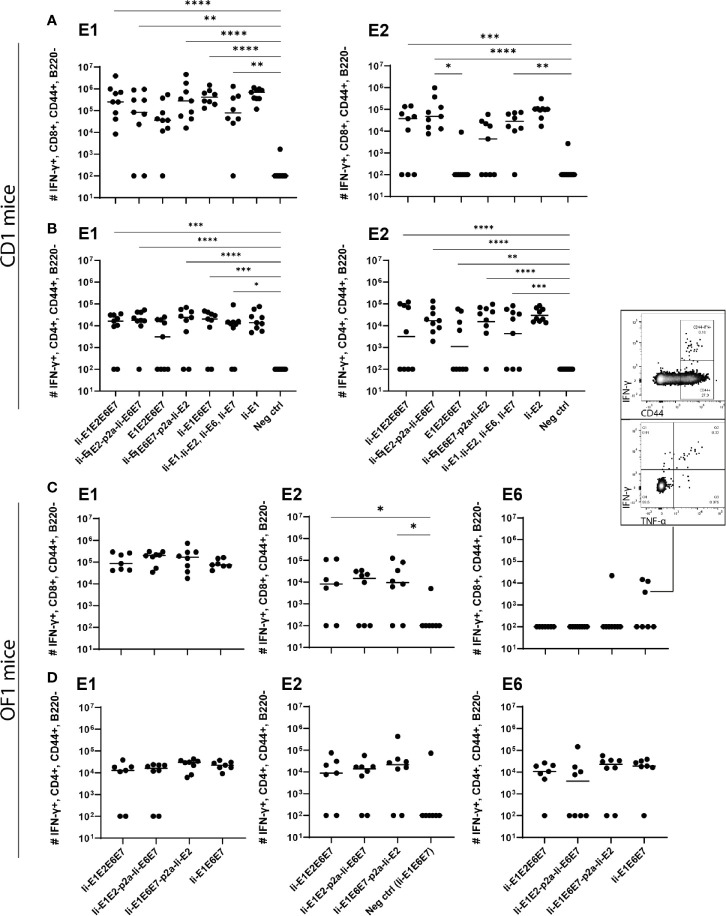
Immunization of mice with adenovirus vectors induces potent cellular immune responses. CD1 mice **(A, B)**, or OF1 mice **(C, D)** were immunized with rAd vaccine (2×10^7^ IFU) encoding the various MfPV3 early antigens as indicated. Mice were sacrificed on day 14, spleens were harvested and CD8^+^ and CD4^+^ T-cell immune responses against E1, E2, E6, and E7 were measured using ICS and flow cytometry. Negative control groups consist of all mice immunized with rAd encoding antigens not matching the peptide pools used for *in vitro* restimulation, respectively. Each symbol represents one mouse; a representative mouse depicted **(C)**, shows a CD44+IFN-γ+ population. The horizontal bar represents the median. Positive control samples (mice vaccinated with rAd containing only the relevant antigen linked to Ii) are not included in the statistical analysis (multiple comparison adjustment). *p < 0.05; **p < 0.01, ***p < 0.005, ****p < 0.001.

Intramuscular immunization of outbred CD1 mice confirmed that the rAd-formulation of the vaccines induced both CD8^+^ and CD4^+^ T cell responses against E1 and E2 antigens (**Figures 5A, B**). Notably, vaccines including Ii showed a trend towards higher magnitude responses than the vaccine not encoding Ii (**Figures 5A, B**: E1E2E6E7 compared to other vaccines). A tendency of Ii-mediated enhancement of responses was observed for both CD4^+^ and CD8^+^ T cell responses for some vaccine configurations, but the difference was only significant for E2-specific CD8^+^ responses induced by Ii-E1E2-p2a-Ii-E6E7 compared to E1E2E6E7 (**Figure 5B**, upper panel). A direct comparison of the CD8^+^ T-cell response against E1 from Ii-E1E2E6E7 and E1E2E6E7 (Mann-Whitney rank-sum) revealed a p-value of 0.06, which is not strictly significant, but strongly indicates a tendency of Ii improving the magnitude of CD8^+^ T-cells against E1. Further, the vaccine without Ii was the only one for which the CD4^+^ and CD8^+^ T-cell responses against E1 was not significantly different from the negative control. This may confirm earlier observations suggesting that Ii can boost T cell responses in the context of adenoviral vaccine delivery. No difference in the quality of the T cell responses was detected, assessed by the MFI values of IFN-γ (**Supplementary Figure 5C**). When assessing the quality by the fraction of double-positive T cells capable of secreting both IFN-γ and TNF-α it is seen that E1E2E6E7 without Ii seemed to be inferior to other vaccines containing Ii (**Supplementary Figure 5D**). Based on this, we decided to focus our onwards efforts on antigen-fusion vaccine designs containing the Ii adjuvant.

Even though E6 and E7 represent small proteins as compared to E1 and E2 (11.9%, 8.1%, 50.8% and 29.2%, respectively, of the total antigen size), it was remarkable that no responses against E6 or E7 could be observed in any experiment testing the vaccines in outbred CD1 mice. As the MHC-type of the founders of the CD1 strain is unknown, and has not been controlled for since the establishment of the strain, we wondered whether there were limitations in terms of MHC-restriction in the CD1 mice we had obtained. Limitations of genetic diversity of outbred mouse models has indeed been described previously and almost all outbred mouse strains except OF1 descent from the same 9 founder mice (51). Hence vaccination of OF1 mice was pursued to possibly detect different and broader immune responses than in CD1 mice. Besides vigorous T-cell responses against E1 and E2 (**Figures 5C, D**), we did indeed see solid CD4^+^ T-cell responses against E6 (**Figure 5D**). A few mice also had CD8^+^ T-cells reacting towards E6, confirming correct *in vivo* processing and immunogenicity of our vaccine constructs. Furthermore, the majority of the IFN-γ^+^ cells were producing TNF-α as well, confirming the activation phenotype. Overall, the rAd19 delivery of the vaccines efficiently induced T-cells against at least three of the four MfPV3 polyprotein antigens tested, and the immunogenicity of the antigens was enhanced by inclusion of Ii.

### Adenoviral Delivery Induces Specific Killing of Target Cells *In Vivo*

**Figure 6:**
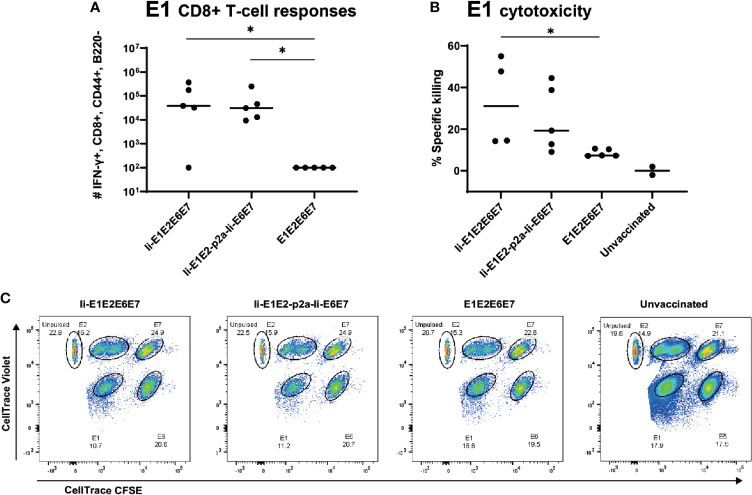
Immune responses in inbred mice and *in vivo* cytotoxicity. Naïve BALB/c mice were immunized with rAd vectored vaccine encoding the indicated MfPV3 early antigens. 14 days post vaccination, immune responses were analyzed by ICS **(A)** or *in vivo* cytotoxicity regarding specific killing of E1-peptide pulsed cells **(B)**. Each symbol represents one mouse. Two unvaccinated mice were included as negative controls. **(C)** Representative plot of *in vivo* cytotoxicity. *p < 0.05.

To assure that the cellular responses induced by the rAd-delivered antigens had cytotoxic capacity, we immunized inbred BALB/c mice with rAds expressing Ii-E1E2E6E7, Ii-E1E2-p2a-Ii-E6E7 and E1E2E6E7, and 14 days later challenged these mice with peptide-pulsed pre-stained syngeneic target cells. As the assay required adoptive transfer of syngeneic target cells, it was necessary to use inbred mice. The vaccines induced CD8^+^ responses against E1 in BALB/c mice (**Figure 6A**), but not against the other antigens (data not shown), whereas CD4^+^ responses were raised against E2 and E6 (**Supplementary Figure 6**). Ii again proved its relevance as molecular adjuvant, as the vaccine without Ii did not give rise to any detectable E1 specific CD8^+^ responses in this inbred mouse model (**Figure 6A**). Consistent with the above, the *in vivo* cytotoxicity assay showed specific killing of E1 labelled cells (**Figures 6B, C**), and the hierarchy of specific killing capability corresponds to the number of IFN-γ^+^ CD8^+^ E1 specific T cells. We can conclude that the designed rAd vaccines induce potent and cytotoxic responses, which supports our hypothesis that this vaccine design will be capable of removing existing MfPV3 infections.

### Ii-Fusion Enhanced Ubiquitination and Proteasomal Degradation

**Figure 7:**
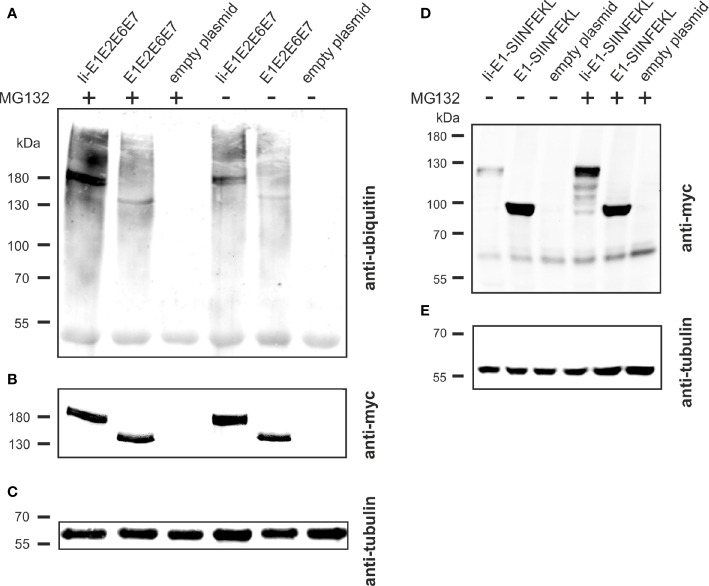
Influence of Ii on ubiquitination and degradation. **(A–C)** pURVac Ii-E1E2E6E7, pURVac E1E2E6E7 or the empty plasmid were transfected into HEK293T cells. 24 h after transfection, cells were treated with MG132 or DMSO as control for 6 h. Myc-tagged proteins were immunoprecipitated and analyzed by western blot using an anti-ubiquitin antibody **(A)** and anti-myc antibody **(B)**. Tubulin levels were monitored using an anti-tubulin antibody as loading control **(C)**. **(D, E)** pURVac Ii-E1-SIINFEKL, pURVac E1-SIINFEKL or empty plasmid were transfected into HEK293T cells. 24 h after transfection, cells were treated with MG132 or DMSO for 6 h. The samples were analysed by western blot using anti-myc antibody **(D)** and anti-tubulin antibody **(E)**.

The molecular basis of the T cell adjuvant effect of Ii has not been fully clarified yet (43), but one of the suggested mechanisms of action is Ii mediated ubiquitination leading to proteasomal degradation of the linked antigen and thereby enhanced MHC-I presentation (**Figure 2C**) (27). To investigate if this mechanism could also account for the enhanced CD8^+^ T-cell responses against the Ii-linked MfPV3 antigens, Ii-E1E2E6E7- and E1E2E6E7-transfected cells were cultivated in absence or presence of the proteasome inhibitor MG132. Anti-ubiquitin western blot analysis of the immunoprecipitated myc-tagged polypeptides revealed stronger ubiquitin-depending signal intensity for Ii-E1E2E6E7 compared to E1E2E6E7 lacking Ii (**Figure 7A**). This effect was even more pronounced when MG132 was present. Myc-specific signals confirmed the fidelity of the immunoprecipitation (IP) procedure, and analysis of the IP supernatants by an anti-tubulin western blot confirmed that comparable amounts of cell lysate were used in the IP procedure (**Figures 7B, C**). A higher level of ubiquitination is commonly associated with faster degradation. This could exemplarily be demonstrated by transiently expressing Ii-E1 and E1, both fused with the C-terminal SIINFEKL peptide, in absence or presence of MG132, respectively (**Figures 7D, E**). Without MG132, Ii-E1-SIINFEKL was hardly detectable whereas a prominent signal could be visualized when MG132 was added. Lower molecular weight signals are indicative of degradation products. This effect was not observed for E1 without Ii, which suggested that Ii induced an accelerated proteasomal degradation, as also reported by Esposito *et al.* (27).

## Discussion

Despite the availability of prophylactic vaccines against HPV infections there is an urgent need for an efficient therapeutic HPV vaccine. Currently, there are different vaccine development approaches focusing either on early stages (15, 22, 50, 52) of cervical lesions or late stages such as invasive cervical cancer (7, 53–56) or on all stages of pre-malignant cervical lesions (57).

*Macaca fascicularis* papillomavirus type 3 (MfPV3) infects cynomolgus macaques and infection is associated with persistence and cervical intraepithelial neoplasia (20, 21). Moreover, MfPV3 is phylogenetically closely related to HPV16 deriving from a shared most recent common ancestor and both viruses possess a similar mechanism of oncogenesis (19). The closely related pathogenesis of MfPV3 in *Macaca fascicularis* and the use of MfPV3 as antigen source, opens a perspective towards testing therapeutic vaccination concepts in naturally infected macaques and evaluate the correlates of protection under almost natural conditions (22).

Here, ten different antigens were designed comprising the viral early proteins E1, E2, E6 and E7 of MfPV3 in different configurations. These constructs combine antigens against early and late stages of papillomavirus persistence and should be capable of inducing T cell responses against both asymptomatic infections, LSIL as well as HSIL and cancers. All antigens were properly expressed, and their expression levels were similar, despite being artificially fused polypeptides.

Notwithstanding the limited immunogenicity of DNA vaccines, pURVac plasmid immunization of CD1 mice allowed for fast immunological evaluation in an outbred mouse model. Based on this initial *in vivo* screening, a refined antigen (Ii-E1E6E7-p2a-Ii-E2, and as reference Ii-E1E6E7) was devised in order to separate E2 from the readthrough protein, and Ii-E6E7E1E2 was excluded for further development of rAd-based vaccine prototypes. Antigen delivery with rAds of serotype 19a/64 confirmed that all antigens were immunogenic, with most of them inducing potent CD4^+^ and/or CD8^+^ T cell responses (**Figure 5**). In general, adenoviral delivery seemed to be overall more immunogenic than DNA vaccination.

Unexpectedly, no responses against E6 and E7 could be measured in these experiments with CD1 mice. Although fewer mice were expected to respond to E6 and E7 as compared to E2 and E1 due to significant differences in size (factor 5) and thus presumably also number of potential epitopes, it was still surprising that none of the 107 outbred CD1 mice in our experiments that were immunized with E6 and/or E7 harboring vaccines were responding. This might be explained by a yet limited number of MHC alleles in CD1 mice. This hypothesis is supported by the fact that this strain descends from only two male and seven female founder mice (51). In a different strain, outbred OF1 mice, we could successfully elicit T-cell responses against MfPV3 E6 confirming the general immunogenicity of this antigen.

Coupling of antigens to certain immune regulatory molecules and T cell adjuvants could be beneficial in terms of breaking the immunosuppressive environment of tumors (53, 58). All immunogens expressed *via* rAds contain the T cell adjuvant Ii (except for E1E2E6E7 which serves as reference), which is known to enhance CD4^+^ and CD8^+^ T cell responses when delivered by viral vectors (25, 27, 43, 59, 60). A trend towards higher magnitudes of responses against E1 and E2 could be observed in outbred mice when vaccinated with rAd Ii-E1E2E6E7 as compared to E1E2E6E7 (**Figure 5**) and, importantly, a significantly higher level of specific killing in animals vaccinated with Ii-adjuvanted vaccines has been shown (**Figure 6**). Whereas enhancement of CD4^+^ T cell response is believed to be induced by trafficking to endolysosomal compartments (43), far less is known about the mechanism of enhancing CD8^+^ T cell response. However, this effect is independent of MHC-II and CD4^+^ T cell response (59), but possibly depending on enhanced MHC-I loading (43). As shown in **Figure 7**, Ii-fusion leads to more pronounced ubiquitination and faster degradation of Ii-E1E2E6E7 as compared to E1E2E6E7 lacking Ii and therefore might increase the number of peptides that can be loaded on MHC I. Such a mechanism has recently also been reported by Esposito *et al.* (27), as a mode of action for the Ii adjuvant to increase the level of antigen fragments available for MHC presentation.

Considering the protein expression pattern during early stages of HPV infection, it is reasonable to assume that potent CD4^+^ and CD8^+^ T cell responses against E1 and E2 should be sufficient to boost naturally raised T cell immunity against PVs in LSIL, especially as E2 responses correlate with absence of lesion progression (14). Noteworthy, E1 is required for successful PV replication in infected cells, and cellular immune responses against E1 have been detected in PBMCs of some patients with HPV^+^ cervical squamous cell carcinoma. The presence of E1 responses strikingly correlated in these patients with improved clinical outcomes, but the responses were of low magnitude, suggesting insufficient APC-mediated activation of T-cells (13). An explanation for this could be that E1 during natural infection, is not abundantly expressed and rapidly degraded through the proteasome as shown for bovine PV (44, 45). Therefore, a vaccine delivery format supporting efficient induction of CD4^+^ and CD8^+^ specific T cells on the fundament of strong expression and efficient antigen processing and presentation is expected to support elimination of premalignant PV transduced cells - provided that early neoplasias display sufficient amounts of E1/MHC-I complexes on their cell surface. Herein, we demonstrated strong expression of E1 comparable to E2, E6 and also E7, probably attributable to the strong CMV promoter-induced overexpression, and additional Ii-induced accelerated proteasomal degradation (**Figure 7**) and MHC-I presentation (**Figure 2C**). Altogether, our E1 containing vaccine constructs gave rise to solid CD8^+^ and CD4^+^ responses, which are cytotoxic to E1-peptide-pulsed target cells (**Figure 6**). This, together with the indication that E1 responses, once induced, are correlated with improved clinical outcome, build a strong case for including E1 in therapeutic HPV vaccine designs.

Our study has limitations regarding weak or absent T-cell responses against E6 and E7 in different mouse strains used in this study. This could potentially be due to immunodominance of E1 and E2 competing with E6 and E7 antigen processing, presentation and induction of specific T-cell responses. Alternatively, we cannot exclude that inactivation of E6- and E7-transforming potential has destroyed relevant epitopes. Ongoing preclinical analysis of viral vector-delivered MfPV3 antigens in macaques and comparable HPV16-derived immunogen designs in selected mouse models will provide further insights regarding the capacity of such immunogens to raise broad T-cell responses to the delivered early antigens including E6 and E7. The latter will also reveal to which extent the antigen design will be transferable to HPV16 and other high-risk HPV types. Given the phylogenetically close relationship and similar mechanisms underlying oncogenesis of the two papillomaviruses (19), similar immunological properties would be assumed.

Taken together, we developed ten immunogens that target the early proteins of MfPV3. These artificially fused polypeptides share similar biochemical and immunological properties without loss of individual antigen responses. Two polypeptides, Ii-E1E2E6E7 and Ii-E1E2-p2a-Ii-E6E7, elicited vigorous T cell responses, in particular, these two antigens were able to induce robust T cell responses against E1, E2 and E6, and to kill peptide-pulsed cells *in vivo*. Due to lower complexity, antigen size and non-inferior immunogenicity, Ii-E1E2E6E7 was chosen for further studies. Ad19a/64 proved to be a suitable vector to deliver Ii-E1E2E6E7 and is currently being validated as a therapeutic vaccine in persistently MfPV3-infected cynomolgus macaques. Moreover, the configuration of this immunogen will serve as template to generate novel vaccine candidates for therapeutic vaccination against high-risk human papillomavirus types.

## Data Availability Statement

The original contributions presented in the study are included in the article/**Supplementary Material**. Further inquiries can be directed to the corresponding authors.

## Ethics Statement

The animal study was reviewed and approved by National Animal Experiments Inspectorate (Dyreforsøgstilsynet, license no. 2016-15-0201-01131).

## Author Contributions

PH, CT and RW designed the study and acquired funding, PN, DB, BA, TW, CP, SS planned and performed the experiments, PN, DB, BA, PH, CT and RW interpreted the data, PN and DB wrote the draft, PN, DB, BA, PH, RW wrote, reviewed and edited the manuscript. All authors contributed to the article and approved the submitted version.

## Funding

This project has received funding from the Eurostars-2 joint programme with co-funding from the European Union Horizon 2020 research and innovation programme (E!12151). The project E!12151 was carried out within the framework of the European funding program “Eurostars” and the German partners were funded by the Federal Ministry of Education and Research. The manuscript reflects only the authors’ view and the European Commission is not responsible for any use that may be made of the information it contains. The funders had no influence on study design, data collection and analysis, decision to publish, or preparation of the manuscript.

## Conflict of Interest

PH is an inventor on a patent detailing the use of the invariant chain as an adjuvant for virally delivered vaccines owned by the University of Copenhagen. The right to use the patent for treating HPV is licensed to InProTher ApS where author PH is the founder, major shareholder, board member and employee. DB is an employed by the company InProTher ApS. TW, CP, and SS are employed by the company SIRION Biotech GmbH. CT is founder and shareholder of SIRION Biotech GmbH and a board member of InProTher ApS. RW is board member of SIRION Biotech GmbH. PN, BA, RW, DB, PH, CP and CT are inventors on a patent application.

## Publisher’s Note

All claims expressed in this article are solely those of the authors and do not necessarily represent those of their affiliated organizations, or those of the publisher, the editors and the reviewers. Any product that may be evaluated in this article, or claim that may be made by its manufacturer, is not guaranteed or endorsed by the publisher.

## Supplementary Material

The Supplementary Material for this article can be found online: https://www.frontiersin.org/articles/10.3389/fimmu.2021.761214/full#supplementary-material
