# Supplementary material for: Design and Immunological Validation of Macaca fascicularis Papillomavirus Type 3 Based Vaccine Candidates in Outbred Mice: Basis for Future Testing of a Therapeutic Papillomavirus Vaccine in NHPs
Source: Front Immunol. 2021 Oct 28;12:761214. doi: 10.3389/fimmu.2021.761214 (PMC8581358; doi:10.3389/fimmu.2021.761214)

Supplementary Material

Supplementary Figure 1. Gating strategy for flow cytometry analysis of pURVac transfected HEK293T cells 48 h post transfection.

Supplementary Figure 2. Gating of spleen samples of a representative animal 14 days after vaccination with rAd-Ii_E1E2E6E7, stimulated with E1 peptide pool. The total number of IFN-γ^+/$CD44^+$/$CD8^+$ or $CD4^+$/$B220^-$ cells reported as the $CD8^+$ and $CD4^+$ responses throughout the article, has been gated this way.

Supplementary Figure 3. (A-D) CD1 mice (5 per group) were immunized 4 times with 0.5 µg DNA of pURVac DNA encoding the indicated MfPV3 early antigens. Mice were sacrificed 7 days post last immunization, spleens were harvested. **(A-B)** $CD8^+$ and $CD4^+$ T-cell immune responses against Ii were measured using ICS and flow cytometry. **(C-D)** The quality of the $CD8^+$ antigen specific cells was assessed by the MFI of IFN-γ and by the fraction of IFN-γ^+ T cells capable of also producing TNF-α. Negative control groups consist of all mice immunized with a pURVac DNA vaccine encoding antigens not covered by the peptide pools used for *in vitro* restimulation. Asterisks between groups indicate significant differences in response-levels after subtraction of background responses. Each symbol represents one mouse.

Supplementary Figure 4. Gating strategy for flow cytometry analysis of rAd-infected A549 cells 48 h post infection.

Supplementary Figure 5. (A-D) CD1 mice were immunized with rAd vectored vaccine (2×10^7 IFU) encoding the various MfPV3 early antigens as indicated. Mice were sacrificed on day 14 and spleens were harvested. **(A-B)** $CD8^+$ and $CD4^+$ T-cell immune responses against Ii were measured using ICS and flow cytometry. **(C-D)** The quality of the $CD8^+$ antigen specific cells was assessed by the MFI IFN-γ and by the fraction of IFN-γ^+ T cells capable of also producing TNF-α. Negative control groups consist of all mice immunized with rAd encoding antigens not covered by the peptide pools used for *in vitro* restimulation. Each symbol represents one mouse. Asterisks between groups indicate significant differences in response-levels after subtraction of background responses.

Supplementary Figure 6. BALB/c mice were immunized with (2×10^7 IFU) rAd vectored vaccine encoding the various MfPV3 early antigens. Mice were sacrificed on day 14, spleens were harvested and $CD4^+$ T-cell immune responses against E2 and E6 were measured using ICS and flow cytometry. Each symbol represents one mouse.

Supplementary Figure 1:

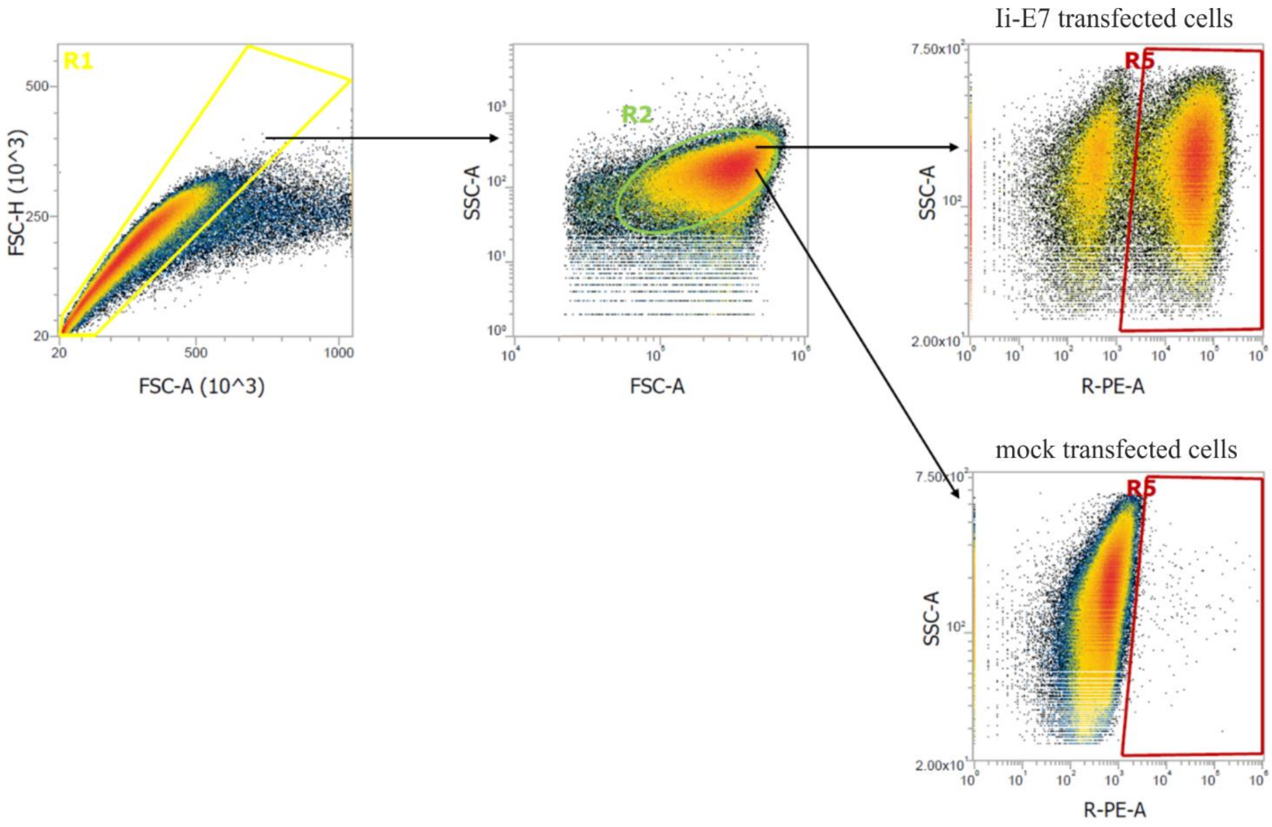

Supplementary Figure 2:

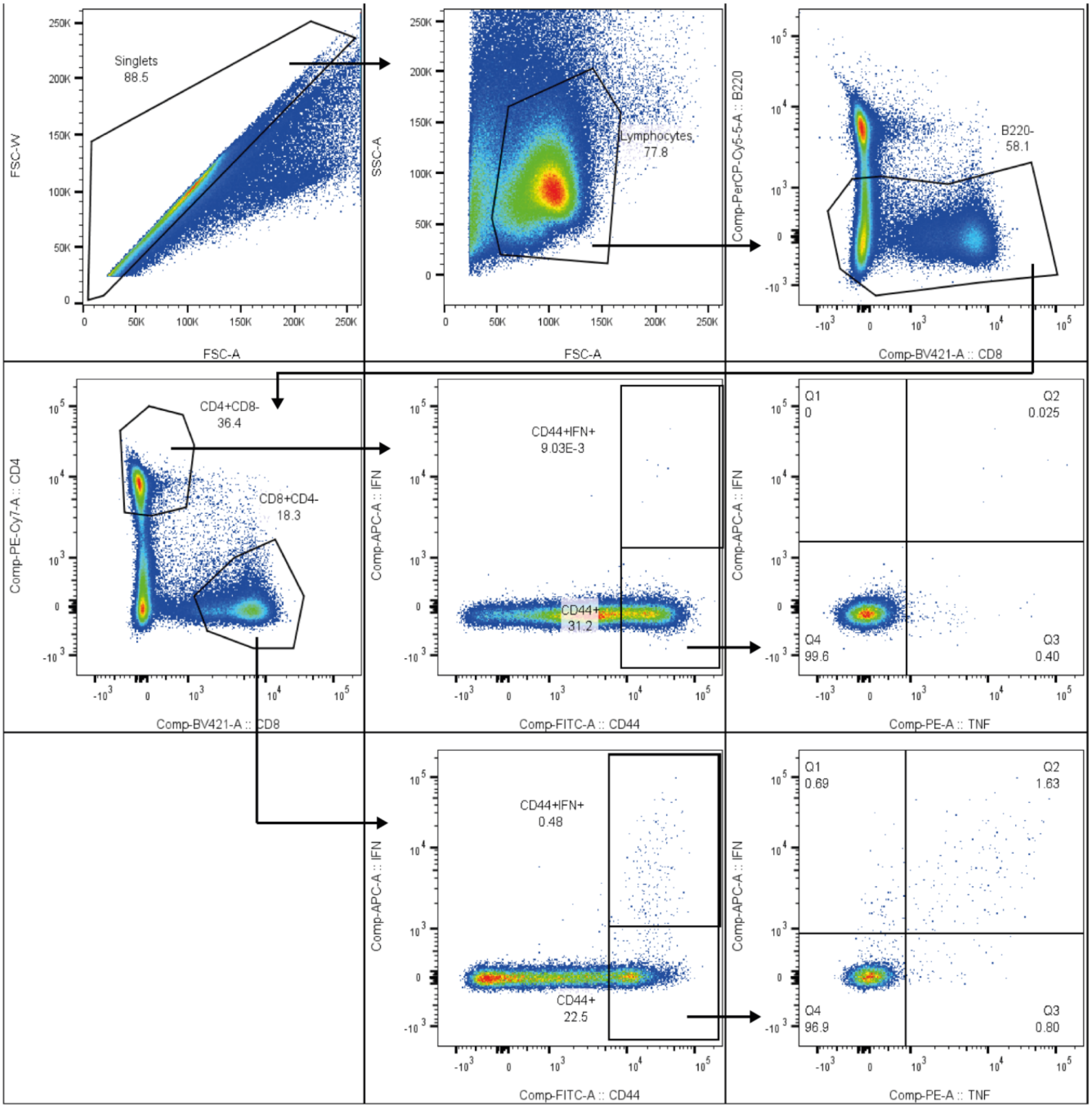

Supplementary Figure 3:

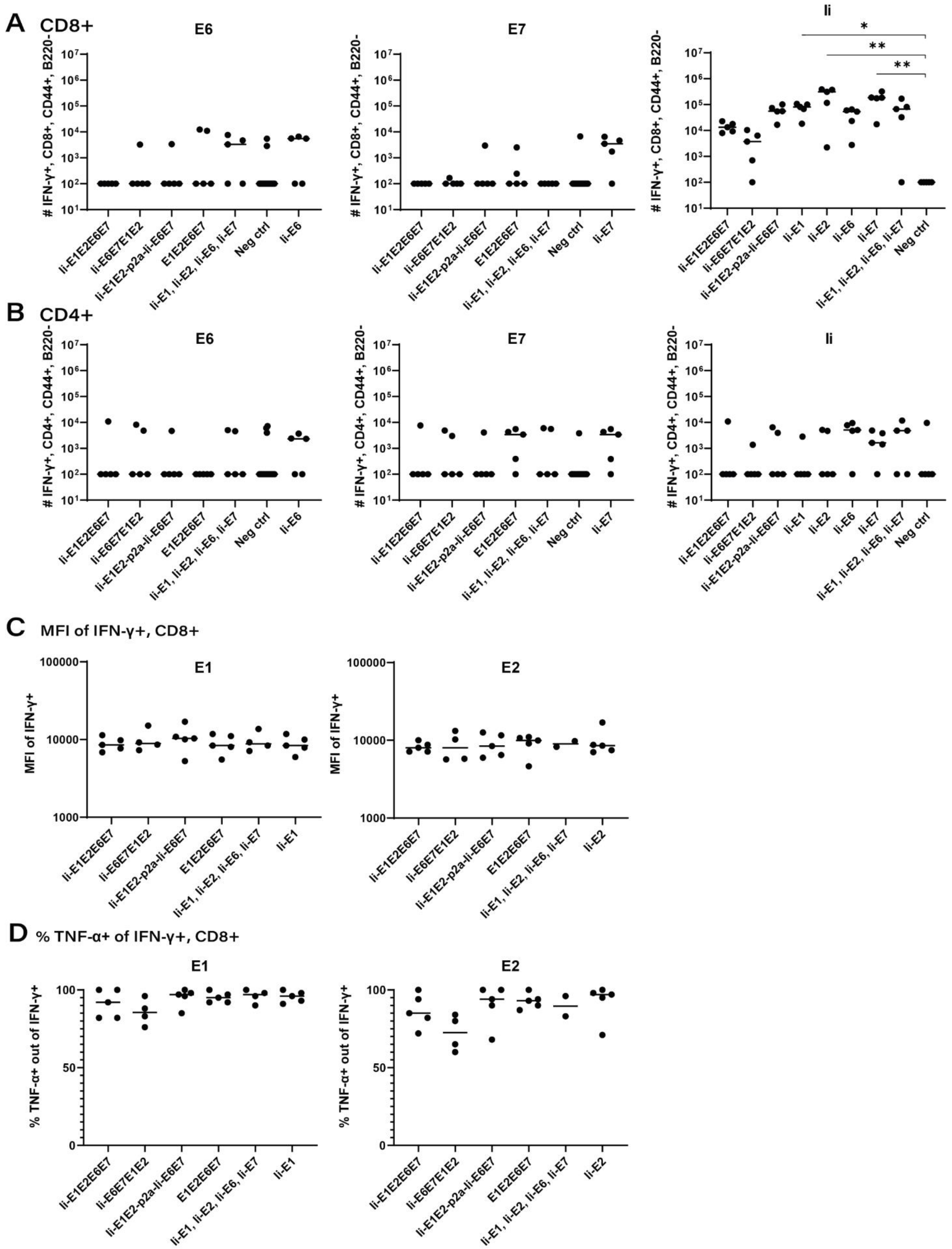

Supplementary Figure 4:

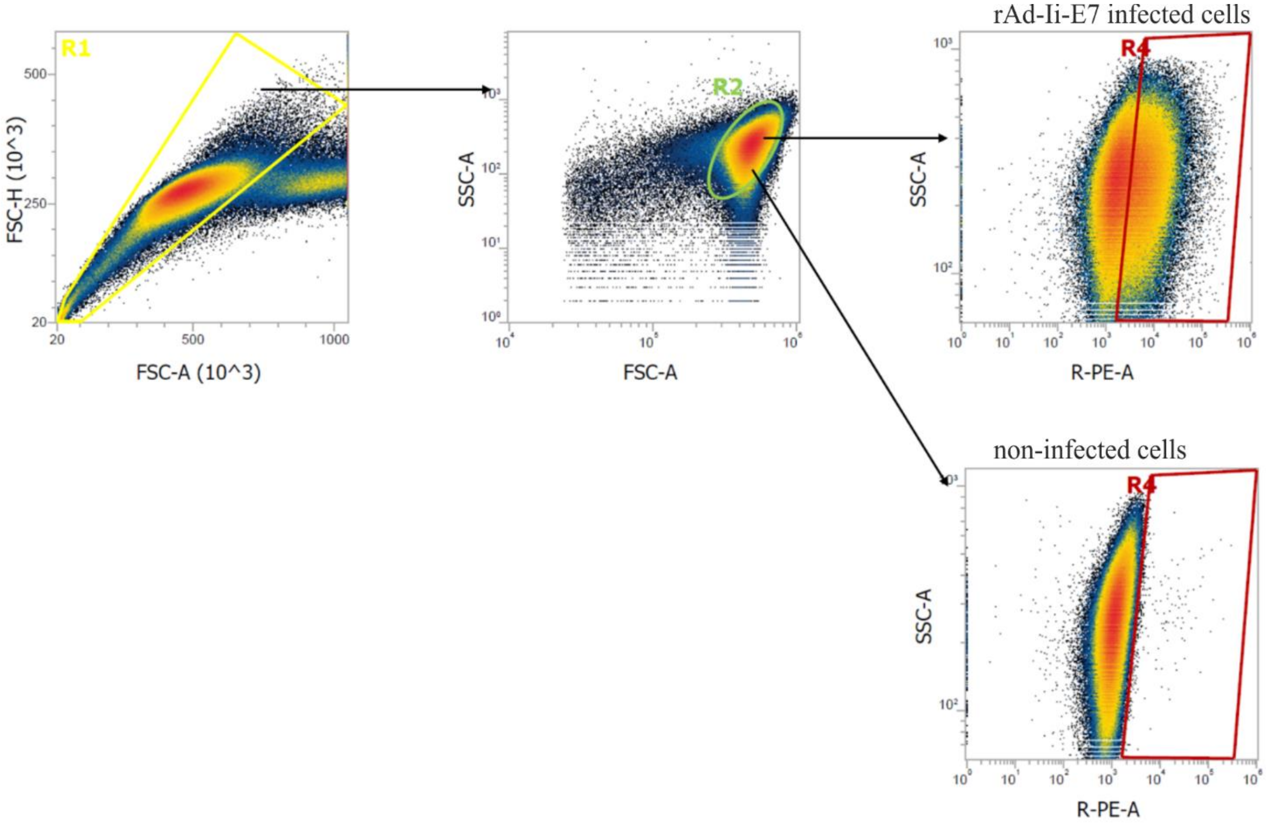

Supplementary Figure 5:

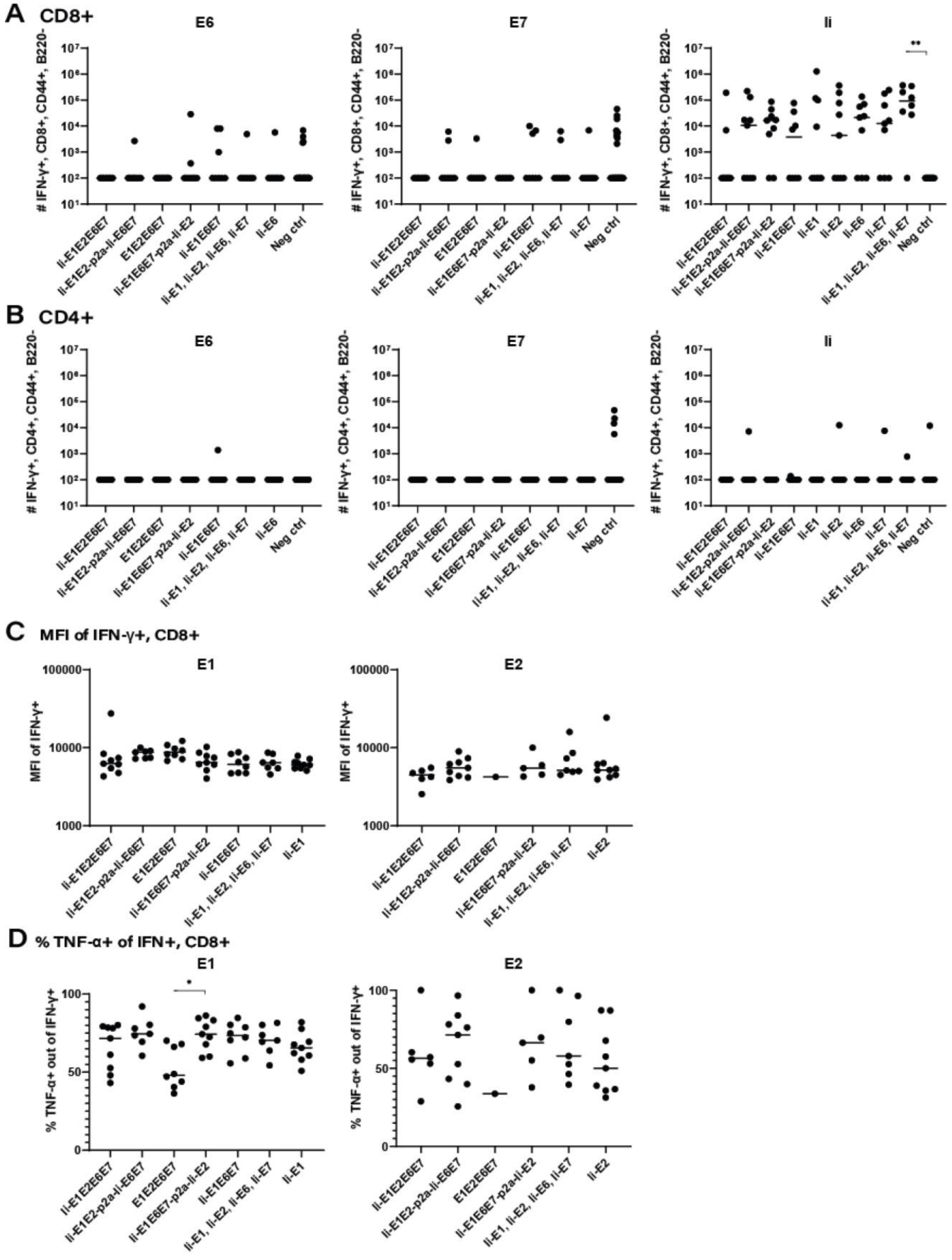

Supplementary Figure 6:

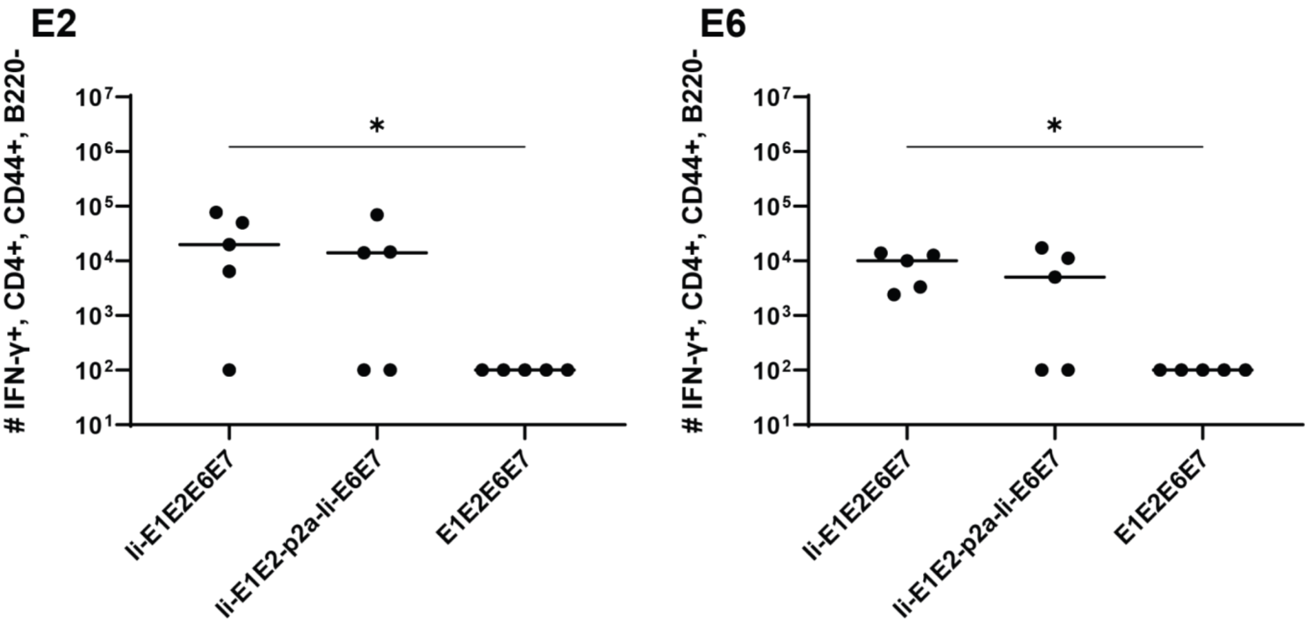

Supplement: Supplementary file 1 [file DataSheet_1.pdf]
